# Design Consideration, Numerical and Experimental Analyses of Mode-Division-Multiplexed (MDM) Silicon Photonics Integrated Circuit with Sharp Bends

**DOI:** 10.3390/s23062965

**Published:** 2023-03-09

**Authors:** Pin-Cheng Kuo, Chi-Wai Chow, Yuan-Zeng Lin, Wahyu Hendra Gunawan, Tun-Yao Hung, Yin-He Jian, Guan-Hong Chen, Ching-Wei Peng, Yang Liu, Chien-Hung Yeh

**Affiliations:** 1 Department of Photonics & Graduate Institute of Electro-Optical Engineering, College of Electrical and Computer Engineering, National Yang Ming Chiao Tung University, Hsinchu 30010, Taiwan; 2 Department of Photonics & Graduate Institute of Electro-Optical Engineering, College of Electrical and Computer Engineering, National Chiao Tung University, Hsinchu 30010, Taiwan; 3 Philips Electronics Ltd., N.T., Hong Kong; 4 Department of Photonics, Feng Chia University, Taichung 40724, Taiwan

**Keywords:** silicon photonics (SiPh), mode-division multiplexing (MDM), Euler bend, non-orthogonal multiple access (NOMA), orthogonal-frequency-division multiplexing (OFDM), optical interconnect

## Abstract

Due to the popularity of different high bandwidth applications, it is becoming increasingly difficult to satisfy the huge data capacity requirements, since the traditional electrical interconnects suffer significantly from limited bandwidth and huge power consumption. Silicon photonics (SiPh) is one of the important technologies for increasing interconnect capacity and decreasing power consumption. Mode-division multiplexing (MDM) allows signals to be transmitted simultaneously, at different modes, in a single waveguide. Wavelength-division multiplexing (WDM), non-orthogonal multiple access (NOMA) and orthogonal-frequency-division multiplexing (OFDM) can also be utilized to further increase the optical interconnect capacity. In SiPh integrated circuits, waveguide bends are usually inevitable. However, for an MDM system with a multimode bus waveguide, the modal fields will become asymmetric when the waveguide bend is sharp. This will introduce inter-mode coupling and inter-mode crosstalk. One simple approach to achieve sharp bends in multimode bus waveguide is to use a Euler curve. Although it has been reported in the literature that sharp bends based on a Euler curve allow high performance and low inter-mode crosstalk multimode transmissions, we discover, by simulation and experiment, that the transmission performance between two Euler bends is length dependent, particularly when the bends are sharp. We investigate the length dependency of the straight multimode bus waveguide between two Euler bends. High transmission performance can be achieved by a proper design of the waveguide length, width, and bend radius. By using the optimized MDM bus waveguide length with sharp Euler bends, proof-of-concept NOMA-OFDM experimental transmissions, supporting two MDM modes and two NOMA users, are performed.

## 1. Introduction

Owing to the high bandwidth demands of broadband applications, such as online gaming, 4K/8K video streaming, internet of things (IOT), etc. [1,2,3,4,5], it is becoming increasingly difficult to satisfy the huge data capacity requirements, since the conventional electrical interconnects suffer significantly from limited bandwidth and huge power consumption. The on-chip optical-interconnect approach emerges as an attractive solution, having the advantages of large bandwidth and low power consumption. Silicon photonics (SiPh) is regarded as a promising technology to significantly enhance the transmission capacity [6,7,8,9,10,11,12]. High performance SiPh devices can be fabricated and manufactured by using complementary metal-oxide-semiconductor (CMOS) fabrication technologies at high yield and low cost. In order to meet the increasing demands of transmission capacity in optical interconnects and optical communication systems, different advanced multiplexing technologies have been utilized, to enhance the spectral efficiency, including wavelength-division multiplexing (WDM) [13,14], polarization-division multiplexing (PolDM) [15,16], and mode-division multiplexing (MDM) [17,18,19], and advanced digital multiplexing schemes, e.g., non-orthogonal multiple access (NOMA) [20,21] and orthogonal-frequency-division multiplexing (OFDM) [22,23]. Among these approaches, MDM is a promising technique in SiPh integrated circuits, to increase the total transmission capacity. A high-performance MDM mode multiplexer (Mux) and de-multiplexer (Demux) can be realized using an asymmetric directional coupler (ADC) [17]. Tbit/s high-capacity transmissions were achieved using ADC-based MDM Mux and Demux [16,19].

In SiPh integrated circuits, waveguide bends are usually inevitable. However, for an MDM system with a multimode bus waveguide, the modal fields will become asymmetric when the waveguide bend is sharp. This will introduce inter-mode coupling and inter-mode crosstalk. To mitigate this issue, various approaches have been proposed. For example, a special waveguide, designed using transformation optics (TO), was proposed, to gradually vary the bus waveguide cross-section profile [24]. Besides, a vertical multimode bus waveguide, supporting single mode and multimode, in the lateral and vertical directions, respectively, was also proposed [25]. However, these schemes require special waveguide heights and are difficult to fabricate. One simple approach to achieve a sharp bend in a multimode bus waveguide is based on a modified Euler curve [16,26]. The Euler bend has a bent section, with the curvature modified gradually; hence, the modes propagating in the straight waveguide section can be converted gradually to the guided modes in the bent section, allowing low-crosstalk transmission. A multimode bus waveguide, having s-shaped sharp bends, based on a modified Euler curve, can support ten guided modes, including four transverse-magnetic (TM)-polarization modes and six transverse-electric (TE)-polarization modes, with a low inter-mode crosstalk of ca. −20 dB having been experimentally demonstrated over a wavelength window of 1520–1610 nm [16].

Although it has been reported in the literature, that sharp bends based on Euler curves allow high performance and low inter-mode crosstalk multimode transmissions, in this work, we discover, by simulation and experiment, that the transmission performance between two Euler bends is length dependent, particularly when the bends are sharp. We investigate the length dependency of the straight multimode bus waveguide between two Euler bends. High transmission performance can be achieved by a proper design of the waveguide length, width, and bend radius. By using the optimized MDM bus waveguide length with sharp Euler bends, proof-of-concept NOMA-OFDM experimental transmissions, supporting two MDM modes and two NOMA users, are performed. For the TE_0_ mode 2-user NOMA signal, data rates of 43.32 Gbit/s and 13.26 Gbit/s are achieved. For the TE_1_ mode two-user NOMA signal, data rates of 44.28 Gbit/s and 13.26 Gbit/s are achieved. All channels fulfill the pre-forward error correction (pre-FEC) requirement (i.e., bit error rate, BER = 3.8 × 10^−3^). Although two MDM modes and one wavelength channel are illustrated here, multiple wavelength operation can be utilized.

## 2. Design and Simulation

**Figure 1:**
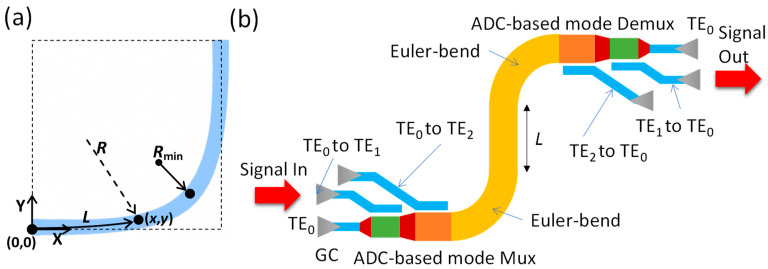
(**a**) Structure of a 90° sharp bend using a Euler curve. (**b**) Schematic of the SiPh integrated circuit, with MDM Mux/Demux and a straight multimode bus waveguide between two Euler bends.

Figure 1a shows the structure of a 90° sharp bend using a Euler curve [16], which is used to reduce the inter-mode coupling. The position (*x*, *y*) of a point in the modified Euler curve (*x*, *y*) is shown in Equation (1). The maximal radius, R_max_, should be large enough to prevent any significant mode mismatch at the junction between the straight multimode bus waveguide and the multimode bent waveguide, while the radius R_min_ should also be large enough to make the waveguide bend adiabatically. *l*_max_ is the total curve length, and *l* is the curve length from the starting point (0, 0) to point (*x*, *y*). Figure 1b illustrates the schematic of the SiPh integrated circuit, including MDM Mux/Demux and a straight multimode bus waveguide between two Euler bends. The MDM Mux/Demux is realized using ADC, which consists of a narrower access waveguide (supporting fundamental TE_0_ mode) and a wider bus waveguide (supporting high-order modes). When the phase-matching condition is satisfied, the fundamental TE_0_ mode can be converted to higher-order modes (i.e., TE_1_, TE_2_, etc.) or vice versa.

**Figure 2:**
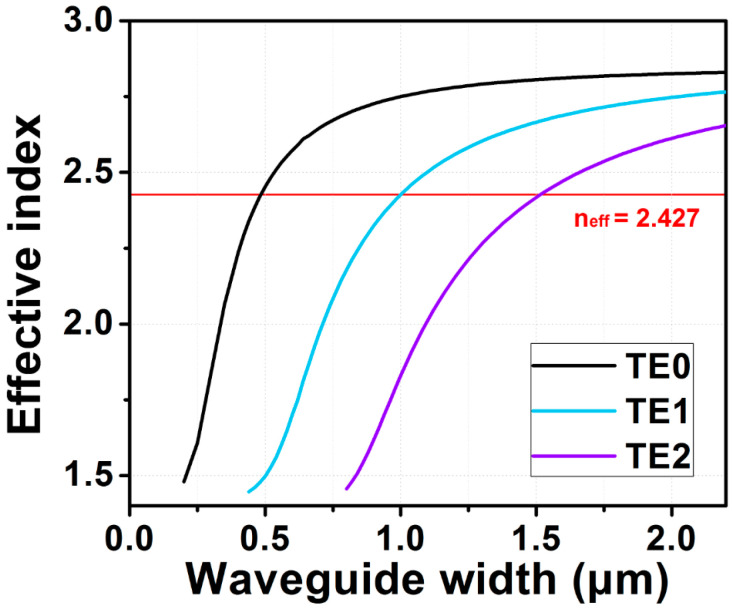
Waveguide effective indices at different waveguide widths for providing phase matching for the MDM Mux/Demux.

The proposed SiPh integrated circuit, shown in Figure 1a, is simulated using the finite-difference time-domain (FDTD) method, in the commercially available software Lumerical^®^. Besides, the devices with different straight multimode bus waveguide lengths, *L*, are also fabricated by IMEC, on a semiconductor-on-insulator (SOI) platform with a waveguide height of 0.22 μm. Figure 2 illustrates the simulated effective indices of the eigenmodes in the SOI waveguide with different core widths. As discussed before, the phase matching condition can be fulfilled at different waveguide widths if they have the same effective refractive indices. As a result, the mode can be down-converted or up-converted between these waveguides. The access waveguide width supporting TE_0_ mode, is 0.35 μm; while the multimode bus waveguide widths supporting the TE_1_, TE_2_, and TE_3_ modes are 0.740 μm, 1.132 μm, and 1.525 μm, respectively. The corresponding coupling lengths are 3.03 μm, 4.32 μm, and 6.17 μm, respectively, as illustrated in Figure 2. The details of the phase-matching condition in the ADC are described in [19].

(1) $$x=A\int_{0}^{l/A}\sin\left(\frac{\theta^{2}}{2}+\frac{A\theta }{R_{\max}}\right)d\theta ;\begin{array}{cc} & y=A\int_{0}^{l/A}\cos\left(\frac{\theta^{2}}{2}+\frac{A\theta }{R_{\max}}\right)d\theta \end{array}\begin{array}{cc} ; & A={\left(\frac{l_{\max}}{\frac{1}{R_{\min}}-\frac{1}{R_{\max}}}\right)}^{\frac{1}{2}} \end{array}$$

## 3. Simulation and Experimental Evaluation of MDM Device with Sharp Euler Bends

**Figure 3:**
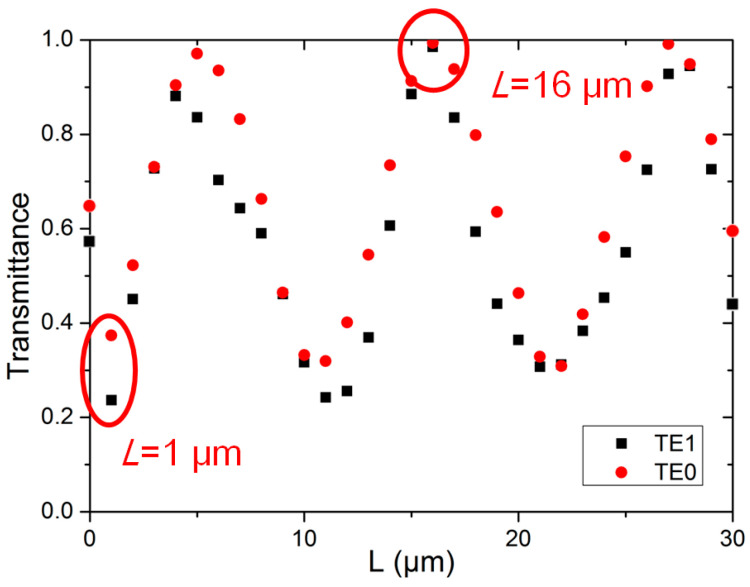
Simulation results of length dependency of a straight multimode bus waveguide between two sharp Euler bends.

Although a Euler bend can allow high-performance and low inter-mode crosstalk multimode transmissions, we discover that the transmission performance is length dependent and periodic in the straight multimode bus waveguide between two sharp Euler bends. Here, the bus waveguide height and width are 0.22 μm and 1.525 μm, respectively. The effective radius, *R_eff_*, of the Euler bend is 12 μm, which is defined by the vertical distance, Y = 12 μm, as shown in Figure 1a. Based on the Euler equation calculation, the horizontal distance is, X = 20.12 μm. The simulation results are shown in Figure 3, a period pattern can be observed in the straight bus waveguide at the fundamental TE_0_ mode and TE_1_ mode.

**Figure 4:**
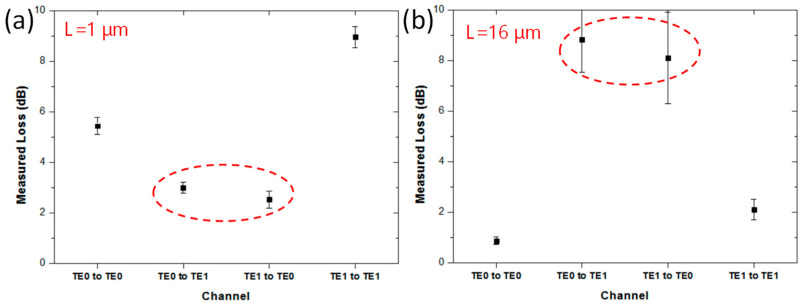
Experimental results of length dependency of a straight multimode bus waveguide between two Euler bends with straight waveguide lengths (**a**) *L* = 1 μm (period pattern trough), and (**b**) *L* = 16 μm (period pattern peak).

We also verified the observed simulation results by experiments. We fabricated ten SiPh integrated circuits, having the structure illustrated in Figure 4a, with straight multimode bus waveguide lengths, *L*, of 1 μm (in five SiPh devices) and 16 μm (in five SiPh devices), respectively, corresponding to the peak and trough transmittances, as shown in Figure 3. The dimensions of the experimental bus waveguide height and width were 0.22 μm and 1.525 μm, respectively. The effective radius, *R_eff_*, of the Euler bend was 12 μm. The dimensions were the same as the simulated device. It is worth pointing out that, as shown in Figure 3, the simulated transmittances at the back-to-back Euler bend (i.e., the straight bus wavelength length between two Euler bends = 0) for TE_0_ and TE_1_, were 0.63 and 0.59 respectively. This means that the crosstalk could be >30% and >40% for the TE_0_ and TE_1_ modes, respectively. Since we only fabricated the device at the transmission maximum and minimum (i.e., periodic pattern peak and trough), the device at the transmission intermediate region was not fabricated.

The periodic pattern in the straight multimode bus waveguide between two sharp Euler bends could be due to the interference of modes. When the optical signal is propagating through the first Euler bend, although the mode conversion using a Euler bend is reduced, when compared to the traditional arc bend, some portions of light at lower-order mode are still converted to higher-order modes. After the transmission of the straight multimode bus waveguide, the higher-order modes are converted back to lower-order modes at the second Euler bend. Due to different group delays of the modes in the straight multimode bus waveguide, the down-converted modes at the second Euler bend beat with the original lower-order mode, producing interference. This will result in the length-dependent periodic pattern.

Figure 4a shows the experimental results at the transmittance trough, when *L* = 1 μm. High average losses, of about 5.44 dB and 8.96 dB, are observed at the TE_0_ mode and TE_1_ mode. Figure 4b shows the experimental results at the transmittance peak when *L* = 16 μm. Low average losses, of about 0.86 dB and 2.1 dB, are observed at the TE_0_ mode and TE_1_ mode. We also experimentally measured the crosstalk between the two MDM modes for the fabricated device. For the poor transmission device (i.e., *L* of 1 μm), the mode crosstalk was high. As shown in Figure 4a, the average TE_0_-to-TE_1_ mode crosstalk is −3.0 dB and the average TE_1_-to-TE_0_ mode crosstalk is −2.52 dB. On the other hand, for the high transmission device (i.e., *L* of 16 μm), the mode crosstalk was low. As shown in Figure 4b, the average TE_0_-to-TE_1_ mode crosstalk is −8.82 dB and the average TE_1_-to-TE_0_ mode crosstalk is −8.1 dB.

**Figure 5:**
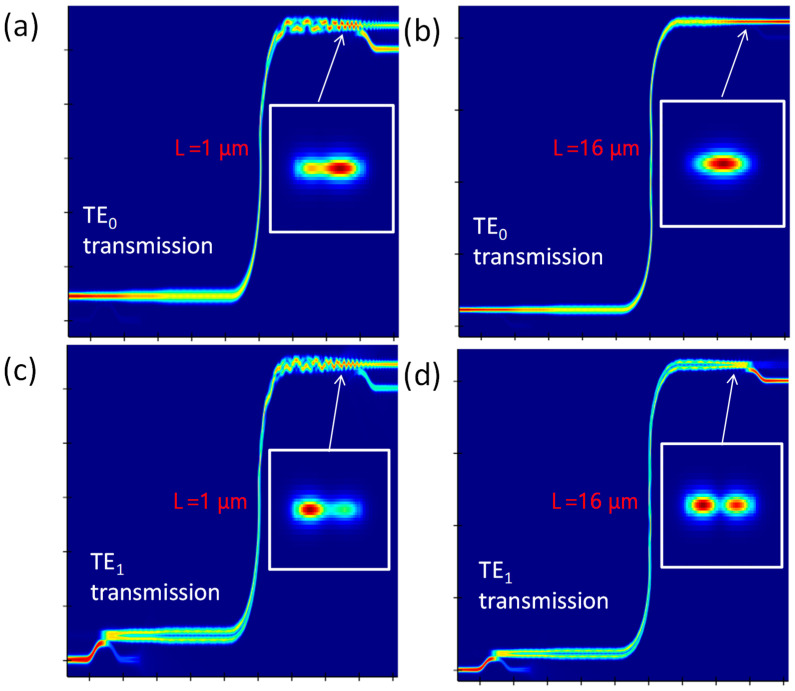
FDTD simulation results of TE_0_ mode transmission at waveguide lengths (**a**) *L* = 1 μm, and (**b**) *L* = 16 μm; and TE_1_ mode transmission at waveguide lengths (**c**) *L* = 1 μm, and (**d**) *L* = 16 μm.

We can also observe that this length dependency could degrade the fundamental TE_0_ mode transmission. Figure 5a–d illustrates the FDTD simulation results from Lumerical^®^, when light is propagating in TE_0_ and TE_1_ modes at straight multimode bus waveguide lengths of 1 μm and 16 μm, respectively. The corresponding mode profiles at different outputs are also displayed. We can observe in Figure 5a, that part of the fundamental TE_0_ mode is converted to the higher TE_1_ mode during the multimode bus transmission. This can explain why a transmittance trough appears in Figure 3. In order to increase the transmittance at the fundamental TE_0_ mode, we can increase the multimode bus waveguide length to 16 μm (i.e., the transmittance peak), or we can decrease the multimode bus waveguide width, to avoid the higher mode conversion. However, decreasing the bus waveguide width will restrict the number of MDM channels; reducing the transmission capacity. Figure 5c,d illustrates the TE_1_ mode transmission at the interference trough and peak, respectively.

In the Lumerical^®^ FDTD simulation, we used the built-in mode monitor function to observe the power percentage of each mode, in order to analyze the mode crosstalk introduced by the Euler bends. Table 1 shows the simulated mode crosstalk of the Euler bend. When the fundamental mode TE_0_ is launched into the device, most power is still preserved at the TE_0_. The power percentages of different modes after a single Euler bend are: TE_0_ = 77.46%, TE_1_ = 21.52%, TE_2_ = 0.87%, and TE_3_ = ~0.00%. When TE_1_ is launched into the device, similarly, most power is still preserved at the TE_1_ mode after a single Euler bend, which is 70.43%, while the power percentages of TE_0_ = 22.27%, TE_2_ = 7.15%, and TE_3_ = 0.01%.

Besides the FDTD simulation, we also performed a theoretical derivation on the effect of the length dependence of the straight multimode waveguide on the transmission of its beating modes. The straight multimode waveguide length-dependent period, Δ*x*, can be calculated using Equation (2), where Δ*θ* is the phase shift, which is equal to 2*π* in this case. *λ* is the wavelength, which is 1.55 μm. Δ*n* is the effective refractive index difference between the beating modes. At this straight multimode bus waveguide, the simulated effective indexes of TE_0_, TE_1_, TE_2_, and TE_3_ modes are 2.79, 2.65, 2.41, and 2.04, respectively.

(2) $$\Delta \theta =\frac{2\pi \Delta n\Delta x}{\lambda }$$

By substituting these values into Equation (2), the theoretical calculated straight multimode waveguide length-dependent period, Δ*x*, between two Euler bends, with *R_eff_* = 12 μm, can be obtained, as shown in Table 2. For example, to calculate the theoretical length period of TE_0_ mode, we calculated that the power ratios of the existing TE_0_, TE_1_, TE_2_, and TE_3_ modes were 77.46%, 21.52%, 0.87% and ~0.00%, respectively, as shown in Table 1. Since the powers of the TE_2_ and TE_3_ modes were small, we calculated the effective refractive index difference between the TE_0_ and TE_1_ beating modes, and Δ*n =* 2.79 − 2.65 = 0.14. As a result, the theoretical calculated straight multimode waveguide length-dependent period was Δ*x* = 11.07 μm, which agrees with our FDTD simulated result. We can also observe in Table 2 that the theoretical calculated and simulated waveguide length periods match with each other.

**Figure 6:**
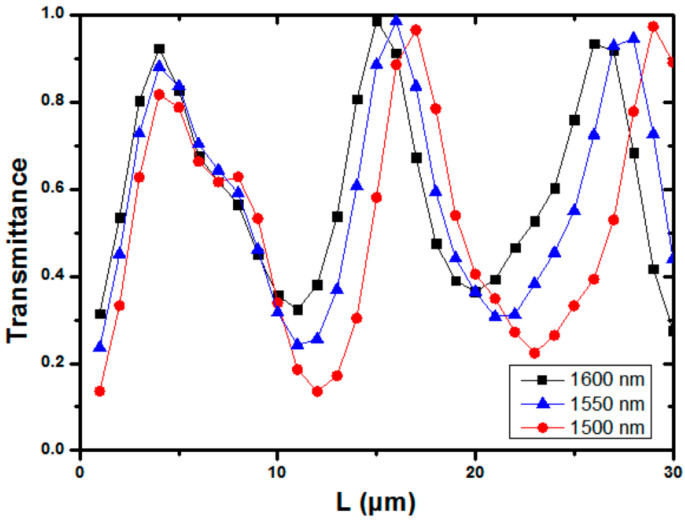
Simulated TE_1_ mode at different wavelengths, with multimode bus waveguide width of 1.525 μm and two Euler bends, with effective radii of 12 μm each.

We also simulated the transmittance of the device at wavelengths of 1500 nm, 1550 nm, and 1600 nm. As different wavelength signals will have different effective indexes in the multimode waveguide, the transmittance is wavelength sensitive. Figure 6 shows the simulated result of TE_1_ mode at different wavelengths, with a multimode bus waveguide width of 1.525 μm and two Euler bends, with effective radii of 12 μm each.

**Figure 7:**
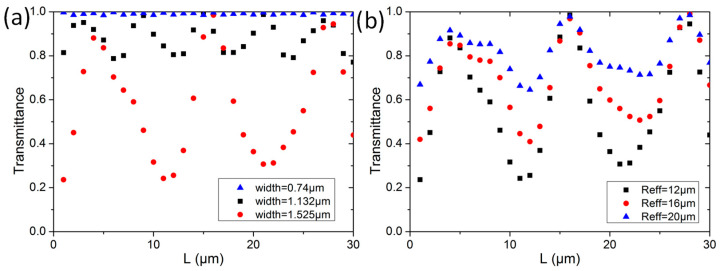
Simulation results of TE_1_ mode (**a**) at different multimode bus waveguide widths and (**b**) at different effective Euler bend radii.

We also investigated other design parameters, to mitigate the establishing of the periodic pattern. As lower-order modes will convert to higher-order modes during the multimode bus waveguide transmission. We can decrease the bus waveguide width. Figure 7a shows the simulation results of TE_1_ mode transmission at effective radius *R_eff_* = 12 μm, with bus waveguide widths of 0.740 μm, 1.132 μm, and 1.525 μm, respectively. We can clearly observe that the periodic pattern disappears at a waveguide width of 0.740 μm, which is the minimum TE_1_ mode supporting waveguide width. However, this will restrict the number of MDM channels and reduce the transmission capacity. Figure 7b shows the simulation results of TE_1_ mode transmission with a bus waveguide width of 1.525 μm, when *R_eff_* is equal to 12 μm, 16 μm, and 20 μm. As expected, the interference effect reduces at a larger bend radius.

## 4. Proof-of-Concept NOMA-OFDM Experiment with Two MDM Modes and Two NOMA Users in the MDM Device with Sharp Euler Bends

**Figure 8:**
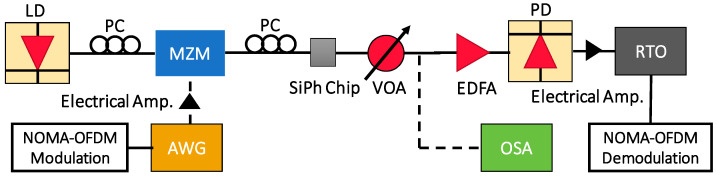
Experimental setup of the proof-of-concept NOMA-OFDM transmission with two MDM modes and two NOMA users, in the MDM device with sharp Euler bends. LD: laser diode; MZM: Mach–Zehnder modulator; AWG: arbitrary waveform generator; VOA: variable optical attenuator; PC: polarization controller; EDFA: erbium-doped fiber amplifier; OSA: optical spectrum analyzer; RTO: real-time oscilloscope; PD: photodiode.

**Figure 9:**
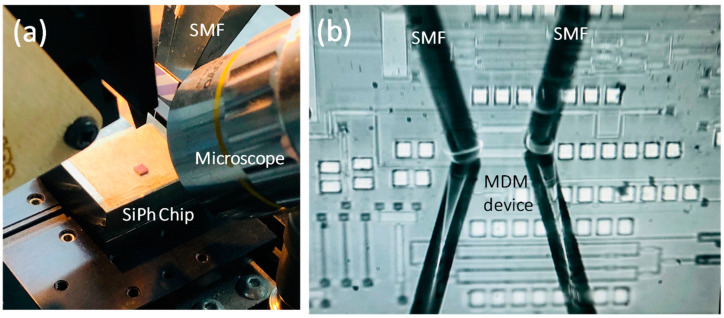
Experimental photos using two SMFs to couple optical signal in and out of the SiPh chip (**a**) before and (**b**) after zoom-in.

Figure 8 shows the experimental setup of the proof-of-concept NOMA-OFDM transmission with two MDM modes and two NOMA users, in the MDM device with sharp Euler bends. The input data include Data_1_ and Data_2_, which are mapped into four quadrature amplitude modulation (QAM). The two data channels, with different powers, are super-positioned in the digital domain to generate the NOMA-OFDM signal via the MATLAB^®^ program. In the experiment, the Data_1_ and Data_2_ are multiplied by different power levels, *P*_1_ and *P*_2_, respectively. After that, the OFDM encoding is executed, involving inverted fast-Fourier-transform (IFFT), parallel-to-serial (P/S) conversion, and cyclic prefix (CP) insertion. Here, the FFT size is 512, subcarrier number is 175, and CP is 32. The produced two-user NOMA-OFDM signal is then applied to a digital-to-analog converter (DAC) to produce a real electrical waveform for the Mach–Zehender modulator (MZM). The DAC is an arbitrary waveform generator (AWG, Tektronix^®^ AWG 70001), having a 20 GHz bandwidth and 50 GS/s sampling rate. The optical NOMA-OFDM signal is produced by the distributed feedback (DFB) laser diode (LD), at a wavelength of 1550 nm, via a 40 GHz MZMs. Then, the optical NOMA-OFDM is launched into the SiPh chip with the MDM device with sharp Euler bends. A polarization controller (PC) is utilized to adjust the TE polarization to the SiPh chip, using standard single-mode fiber (SMF) via a grating coupler (GC). It is worth mentioning that, in the experiment, the TE_0_ mode is launched into the device via the GC, while the TE_1_ mode is launched into the device via the ADC. As illustrated in Figure 1b, the ADC at the input port acts as the mode converter, converting the TE_0_ mode into TE_1_ mode for the bus multimode waveguide. After the multimode waveguide and the two Euler bends, the TE_1_ mode will be converted back to TE_0_ mode at the output port, for the measurement. In this proof-of-concept demonstration, the two NOMA channels at one mode are launched simultaneously; however, the two MDM modes are launched separately into the device, since the fiber ribbon alignment is not available. Figure 9a,b illustrates the experimental photos using two SMFs to couple optical signal in and out of the SiPh chip, before and after zoom-in. Based on the numerical and experimental studies in Section 3, the MDM device used here has two sharp Euler bends and straight multimode bus waveguide length, *L*, of 16 μm, corresponding to the peak transmittance, as shown in Figure 3.

After propagating through the MDM device, the NOMA-OFDM signal is coupled out via SMF and GC. A variable optical attenuator (VOA) and an erbium-doped fiber amplifier (EDFA) are utilized, to adjust and amplify the received optical power, for BER evaluation. The NOMA-OFDM signal received by a photodiode (PD) is captured by a real-time oscilloscope (RTO, LeCroy^®^ 816ZI-B) for analog-to-digital conversion (ADC). The RTO has a 16 GHz analog bandwidth and 80 GS/s sampling rate. The NOMA-OFDM decoding is executed via Matlab^®^ and LabVIEW^®^ programs. It includes serial-to-parallel (S/P) conversion, channel estimation, and FFT. To digitally demultiplex the two NOMA signals, successive interference cancellation (SIC) is employed [23]. The first step of the SIC process is to estimate the gain from the channel response, to decide the decoding sequence, in which the NOMA channel with higher power will be decoded first, while the other NOMA channels can be considered as noise. In this work, Data_2_ has a higher power than Data_1_; hence, it will be decoded first. Then, the estimated signal is remodulated and multiplied by the channel response, before subtracting it from the total NOMA-OFDM signal. After this, the second channel Data_1_ can be retrieved.

**Figure 10:**
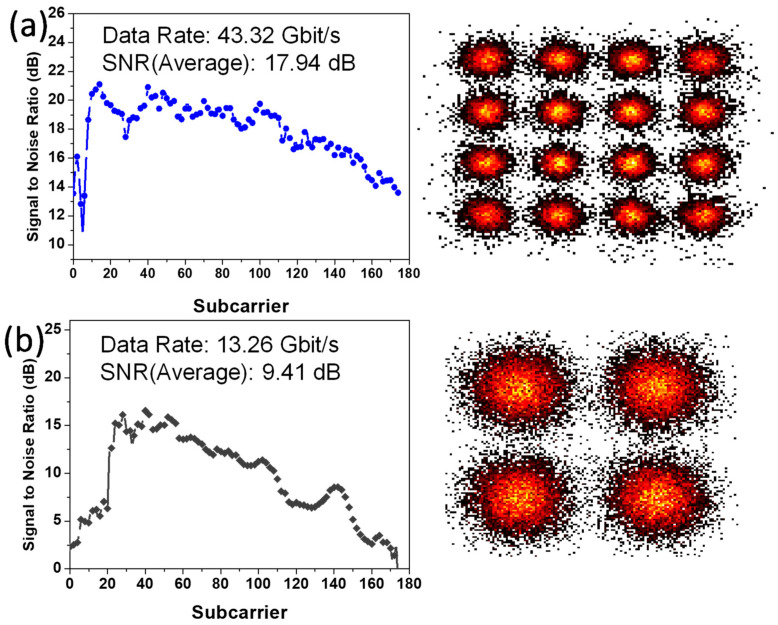
Measured SNRs of NOMA (**a**) Data_2_ and (**b**) Data_1_ over all the 170 OFDM subcarriers at MDM TE_0_ mode, with corresponding constellation diagrams of the NOMA Data_2_ and Data_1_.

**Figure 11:**
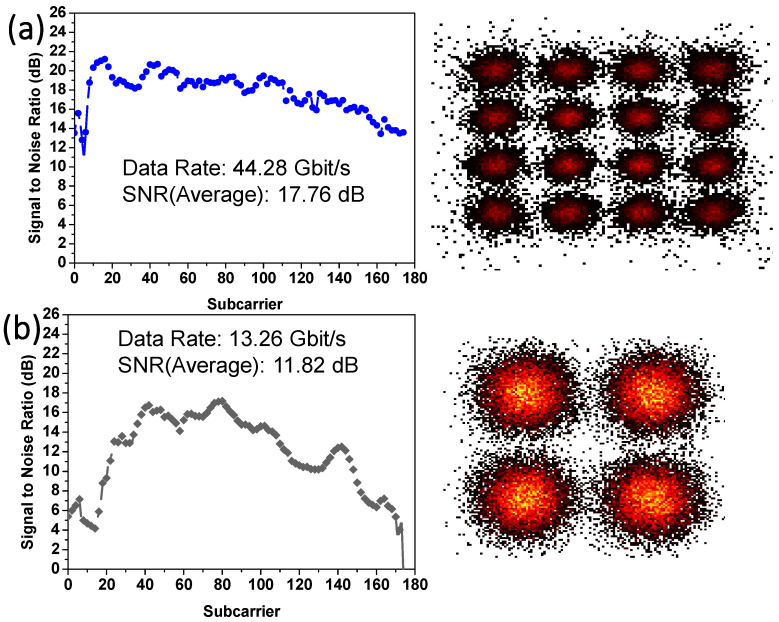
Measured SNRs of NOMA (**a**) Data_2_ and (**b**) Data_1_ over all the 170 OFDM subcarriers at MDM TE_1_ mode, with corresponding constellation diagrams of the NOMA Data_2_ and Data_1_.

The NOMA-OFDM transmission includes two MDM modes and two NOMA channels, at the single wavelength of 1550 nm; hence, there are four data channels in total. Figure 10a,b shows the experimental signal-to-noise ratios (SNRs) of NOMA Data_2_ and Data_1_ over all the 170 OFDM subcarriers at MDM TE_0_ mode. The corresponding constellation diagrams of the NOMA Data_2_ and Data_1_ are also included. NOMA Data_2_ has a higher power, with an average SNR of 17.94 dB, achieving a data rate of 43.32 Gbit/s and BER of 1.1 × 10^−3^. NOMA Data_1_ has a lower power, with an average SNR of 9.41 dB, achieving a data rate of 13.26 Gbit/s and BER of 1.5 × 10^−3^. Figure 11a,b shows the experimental SNRs of NOMA Data_2_ and Data_1_ over all the 170 OFDM subcarriers at MDM TE_1_ mode. Similarly, the corresponding constellation diagrams of the NOMA Data_2_ and Data_1_ are also included. NOMA Data_2_ has a higher power, with an average SNR of 17.76 dB, achieving a data rate of 44.28 Gbit/s and BER of 1.07 × 10^−3^. NOMA Data_1_ has a lower power, with an average SNR of 11.82 dB, achieving a data rate of 13.26 Gbit/s and BER of 1.27 × 10^−3^. All the four channels can satisfy the pre-FEC requirement (BER = 3.8 × 10^−3^). It is worth noting that, although two MDM modes and one wavelength channel are illustrated in this proof-of-concept demonstration; the proposed analysis can be scaled to higher-order modes and multiple wavelength operation.

## 5. Conclusions

SiPh is considered as a promising technology to increase the optical interconnect capacity, while decreasing the power consumption. Combining WDM, MDM, NOMA, and OFDM at the same time, can significantly increase the transmission capacity for the SiPh optical interconnects. Waveguide bends are inevitable in SiPh integrated circuits, and it has been reported in the literature, that sharp bends, using Euler curves, allow low inter-mode crosstalk multimode transmissions. In this work, we discovered, by simulation and experiment, that the transmission performance between two Euler bends is length dependent, particularly when the bends are sharp. We investigated the length dependency of the straight multimode bus waveguide between two Euler bends. High transmission performance can be achieved by a proper design of the waveguide length, width, and bend radius. For example, when the bus waveguide height and width were 0.22 μm and 1.525 μm, respectively; and the effective radius of the Euler bend was 12 μm, a periodic length dependent interference pattern was observed. High transmission was observed at straight multimode bus waveguide lengths of 6 μm and 16 μm; while low transmission was observed at bus waveguide lengths of 1 μm and 11 μm. The experimental results agreed with the simulation. By using the optimized MDM bus waveguide length of 16 μm, with sharp Euler bends, a proof-of-concept NOMA-OFDM experimental transmission supporting two MDM modes and two NOMA users was performed. For the MDM TE_0_ mode transmission, the NOMA channels Data_2_ and Data_1_, had average SNRs of 17.94 dB and 9.41 dB, respectively, achieving data rates of 43.32 Gbit/s and 13.26 Gbit/s. For the MDM TE_1_ mode transmission, the NOMA channels Data_2_ and Data_1_, had average SNRs of 17.76 dB and 11.82 dB, respectively, achieving data rates of 44.28 Gbit/s and 13.26 Gbit/s. All the four channels satisfied the pre-FEC requirement.

## Figures and Tables

**Table 1:** Simulated mode crosstalk of the Euler bend.

	Mode Crosstalk	TE_0_	TE_1_	TE_2_	TE_3_
Input Mode					
TE_0_		77.46%	21.52%	0.87%	~0.00%
TE_1_		22.27%	70.43%	7.15%	0.01%
TE_2_		0.18%	0.75%	88.16%	3.67%
TE_3_		0.02%	0.07%	3.78%	93.48%

**Table 2:** Theoretical calculated and simulated waveguide length period.

Mode	Theoretical Calculated (μm)	Simulated (μm)
TE_0_	11.07	11
TE_1_	11.07	11
TE_2_	6.45	6.25
TE_3_	4.19	4.16

## Data Availability

The data presented in this study are available from the first author upon request.
